# A qualitative framework-based evaluation of radiology clinical decision support initiatives: eliciting key factors to physician adoption in implementation

**DOI:** 10.1093/jamiaopen/ooz002

**Published:** 2019-02-22

**Authors:** Laura Haak Marcial, Douglas S Johnston, Michael R Shapiro, Sara R Jacobs, Barry Blumenfeld, Lucia Rojas Smith

**Affiliations:** 1 Digital Health and Clinical Informatics (DHCI) Division of eHealth, Quality and Analytics (eQUA), RTI International, Rockville, MD, USA; 2 Digital Health Policy & Standards, RTI International, Waltham, MA, USA; 3 Digital Health & Clinical Informatics, RTI International, Research Triangle Park, NC, USA; 4 Social & Health Organizational Research & Evaluation, RTI International, Research Triangle Park, NC, USA; 5 Social & Health Organizational Research & Evaluation, RTI International, Washington, DC, USA

**Keywords:** radiology, clinical decision support, HCIA, evaluation, qualitative

## Abstract

**Objectives:**

To illustrate key contextual factors that may have effects on clinical decision support (CDS) adoption and, ultimately, success.

**Materials and Methods:**

We conducted a qualitative evaluation of 2 similar radiology CDS innovations for near-term endpoints affecting adoption and present the findings using an evaluation framework. We identified key contextual factors between these 2 innovations and determined important adoption differences between them.

**Results:**

Degree of electronic health record integration, approach to education and training, key drivers of adoption, and tailoring of the CDS to the clinical context were handled differently between the 2 innovations, contributing to variation in their relative degrees of adoption and use. Attention to these factors had impacts on both near and later-term measures of success (eg, patient outcomes).

**Discussion:**

CDS adoption is a well-studied early-term measure of CDS success that directly impacts outcomes. Adoption requires attention throughout the design phases of an intervention especially to key factors directly affecting it, including how implementation across multiple sites and systems complicates adoption, which prior experience with CDS matters, and that practice guidelines invariably require tailoring to the clinical context.

**Conclusion:**

With better planning for the capture of early-term measures of successful CDS implementation, especially adoption, critical adjustments may be made to ensure that the CDS is effectively implemented to be successful.

## Introduction

Clinical decision support (CDS) tools have shown improvements in efficiency and accuracy for medication ordering by reducing medical errors, improving formulary adherence, and improving patient outcomes through better management of contraindications and allergic events.^1^ Since the introduction of the Meaningful Use (MU) program, greater attention is being paid to CDS components of the electronic health record (EHR). Future stages of MU (now the Quality Payment Program) will require the integration of CDS for radiology exams.^2^ The CDS tools give health care providers specific recommendations on the optimal order for selecting and conducting imaging tests, such as X-rays or magnetic resonance imaging. Although many articles in the literature describe the successes or failures of specific CDS initiatives,^1^^,^^3^^,^^4^ some provide key factors in ensuring successful CDS adoption and implementation. This work is a detailed comparison of 2 similar radiology CDS initiatives, which, despite their differing contexts, encountered many of the same hurdles in design and implementation in ensuring successful adoption.

The success of health information technology (HIT) adoption in clinical environments and by health care providers depends on appropriate management in the contexts in which such innovations are implemented. Contexts can differ by the number of EHR systems and implementation sites involved, types of clinical settings, and key stakeholders’ experiences. Identifying which elements of adoption and implementation in light of different contexts give rise to success and sustainability of an initiative can be valuable when deciding next steps in disseminating innovative health care solutions.

In this work, we highlight the relevant factors that influenced adoption of 2 radiology CDS interventions in 2 care contexts. Our specific research aims were as follows:

1. Examine specific framework elements and related constructs that affected adoption.
2. Of those characteristics, describe the ones that had the greatest effect on adoption for the 2 awardees.

## MATERIALS AND METHODS

In 2012, the Centers for Medicare & Medicaid Services (CMS) awarded $162 622 080 to 24 health care organizations to demonstrate impacts on health care quality, cost, and outcomes over a 3-year period. Established as part of the Health Care Innovation Awards (HCIA) (https://innovation.cms.gov/initiatives/Health-Care-Innovation-Awards/) for Community Resource Planning, Prevention, and Monitoring, these awardees were very diverse in the types of organizations represented and the focus and scale of their interventions. Some tested processes and tools to improve the coordination of care across multiple health care settings, while others tried to improve patient care through innovative HIT, decision support tools, or changes to the composition of the health care workforce. In an effort to identify and understand the models that can be replicated on a broader scale, the Center for Medicare & Medicaid Innovation (CMMI) contracted with RTI International to evaluate the 24 HCIA Community Resource awardees (HCIA awardees). Here, we provide a detailed qualitative analysis of 2 radiology CDS awardees included in the RTI evaluation.

Of all of the initiatives evaluated, these 2 were strikingly similar in their approach to the problem, their proposed solution, their intended impacts, and their plans for measuring outcomes. Moreover, the methodologies of the 2 innovations were the same in several ways including proposing to transform existing guidelines into usable CDS, taking somewhat of a user-centered design approach to CDS development, plans to integrate into the existing EHR environments, and plans regarding the training of clinicians to use the tool.

To complete this analysis, we used qualitative research methods to describe the similarities and differences in factors affecting adoption of 2 radiology CDS interventions one offered by Altarum Institute and the other offered by Imaging Advantage (IA). Both awardees designed and implemented radiology-based CDS software tools linked to EHR systems. These 2 institutions’ interventions were granted Health Care Innovation Awards (HCIA) for the period from January 2014 through December 2015. The settings of the 2 awardees—for Altarum the radiology CDS targeted physician practices, for IA, the radiology CDS was used in emergency departments (EDs)—were different, which required tailored approaches to elements of the innovations, especially the implementation process. However, these differences were well understood by the awardees and successful approaches in these contexts are well documented in the literature.^5^ IA implemented its CDS innovation in the EDs of 4 Tenet Healthcare hospitals in the Chicago area. Altarum worked with 2 large outpatient practice partner organizations, McLaren Physician Partners (MPP) and United Physicians (UP) with employed and independent physicians in Michigan.

The process redesign framework,^6^ developed from the Consolidated Framework for Implementation Research (CFIR),^7^^,^^8^ was used to organize, analyze, and compare qualitative data. The CFIR has been used widely to evaluate health IT implementations and has focused on decision support in many cases.^9–11^ As a broad, overarching framework, the CFIR is particularly useful in making comparisons across different contexts and in generalizing about different implementation experiences.^12–18^ A systematic review by Kirk et al in 2016 suggests the following be adhered to in ensuring the most successful application of the CFIR: (1) Consider how to most meaningfully use it, (2) Report how CFIR constructs were selected and used, (3) Assess the association of CFIR constructs with outcomes and (4) Integrate the CFIR into the entire research process. For this post hoc deductive analysis using the CFIR, steps 1 and 2 were easy to meet but steps 3 and 4 were not possible given the nature of the evaluation.

Elements of the CFIR framework used to focus this evaluation included: intervention characteristics, characteristics of individuals and teams, and the process of implementation. We also include a brief discussion of the measures of implementation, outcomes, and inner and outer setting (internal and external contextual factors). We focused on comparing physician adoption between the 2 awardees along these framework elements.

We used data from stakeholder interviews and a survey of health care providers (both were IRB approved, full survey details are here: https://downloads.cms.gov/files/cmmi/hcia-communityrppm-secondevalrpt.pdf) to describe how contextual factors influenced the adoption of CDS tools used in radiology. The provider survey was administered across all innovations. For Altarum, 460 invitations to complete the survey were sent. For IA, 64 invitations to complete the survey were sent. Sample survey questions are included in Supplementary Appendix A.

Interviews conducted either in person as part of a site visit or over the phone (virtual site visit) were guided by a semi-structured interview guide (see Supplementary Appendix B for sample interview questions). Between 8 (Altarum) and 15 (IA) individuals from each innovation organization and/or affiliated partners or sites were interviewed. Interviewee types included the project management team responsible for design and implementation of the intervention, at least one member of the technical development team (CDS software development) and at least one member of an evaluation team. Site administrators were also interviewed, including medical directors (ED), radiology directors, practicing radiologists and generalists, some nurse practitioners and/or physician assistants, and other members of the implementation team at several of the practice sites for both interventions.

Interview data and progress reports were deductively coded in NVivo 11 by a subset of the interviewers. To ensure high inter-rater reliability (>85%) for this evaluation, 2 analysts independently and concurrently coded a subset (20%) of data (eg, interview notes, progress reports). When they were finished, the qualitative task manager used NVivo to run a coding comparison report to identify any codes with weak (<85%) agreement. The task manager adjudicated disagreements when agreement was below the project threshold, and the debriefing meetings provided opportunities to review and refine the codes in question. Weak agreement among coders diminished as the evaluation continued, given convergence in skill and knowledge among members of the coding team.

Coded reports of qualitative data were reviewed to specifically identify innovation framework elements related to successful adoption in terms of reach (as specified by each awardee when applying for HCIA funding). The manuscript team then used an inductive process to elicit framework element constructs that had more influence on successful adoption than others. Reports were extracted and reviewed along the framework elements and constructs outlined in Table 1. The study team re-reviewed the coding reports to elicit specific comparisons between these 2 awardees in terms of provider adoption of the radiology CDS tools.

**Table 1.** Mapping framework elements to constructs as identified in the inductive analysis

Element in process redesign framework	Critical construct (identified during inductive process)
Intervention characteristics^a^	Evidence strength and quality, adaptability, trialability and compatibility, workflows
Process of implementation^a^	Goal setting, champions, engaging and executing, reflecting and evaluating, measurement capability and data availability
Measures of implementation^a^	Acceptability, adoption, and abandonment; reach and replicability; sustainability; penetration and evolvability
Outer setting	External networks, technological environment
Inner setting	Implementation climate
Characteristics of individuals and teams^a^	Role, skills and competencies, and collective efficacy
Outcomes	Effectiveness and efficiency, cost

a Focus areas for this research.

## RESULTS

We used both the provider survey results and the interview analysis to make meaningful comparisons between the awardees and identify specific targets as key factors for adoption. The survey elicited feedback from a broad group of providers including family and internal medicine physicians for Altarum and a mix of physicians and mid-level providers, such as physician assistants, for IA. For Altarum, 95 providers completed the survey, 57 were ineligible, resulting in a response rate of 23.6%. For IA, 18 providers completed the survey, 8 were ineligible, resulting in a response rate of 32.1%. Of the broader set of HCIA awardees, these were by far the lowest rates of response to the provider survey. Providers generally were not very satisfied with the 2 imaging CDS implementations. The majority of imaging-focused innovation providers (53.0%) reported being either moderately or slightly satisfied with the innovations, whereas only 27.7% reported being extremely or very satisfied with the innovation. In addition, a significant proportion of imaging respondents either strongly or somewhat disagreed that the innovation saved them time (42.1%) and strongly or somewhat agreed that the logistics of the innovation were a burden on them and their staff (44.2%).

The fundamental needs of both providers and patients and the basic goals for these 2 imaging CDS implementations were largely the same: to address issues of quality, overprescribing (hence health care costs), and potential patient harm (radiation exposure), and to be prepared for a mandated requirement regarding the use of CDS for imaging decisions. Table 2 provides a mapping of results according to framework elements and corresponding constructs for each awardee.

**Table 2.** Mapping of results according to framework elements and corresponding constructs for each awardee

Framework element/construct	Both	Altarum	Imaging advantage
Outer setting			
External networks	Altarum and IA approached the problem of appropriate cost and quality of radiological imaging selection with clinical decision support aimed at general practice clinicians	Altarum worked with 2 large outpatient practice partner organizations, McLaren Physician Partners (MPP) and United Physicians (UP) with employed and independent physicians in Michigan	IA implemented its CDS innovation in the EDs of 4 Tenet Healthcare hospitals in the Chicago area
Technical environment	The preferred technical environment for the CDS solutions for both Altarum and IA was either within the EHR or tightly coupled with the EHR	Technical partners for Altarum engaged early on with UP but were less established, resulting in limited EHR integration for initial launch	Technical partners for IA (medCPU and Tenet hospitals IT) were well established
Clinical environment	The choice of clinical environment for Altarum and IA came down to existing partner organizations	Altarum, because of the decentralized structure of its partner organizations (UP and MPP), struggled to find a willing and widely used EHR partner	For IA, working within the Tenet hospital EDs provided a specific context for technical integration, training, and rollout
Inner setting			
Implementation climate	Working with end users was largely done through third party organizations for both Altarum and IA	Altarum largely used its partners to establish a connection with the provider end users of ImageSmart	With each change, IA and medCPU coordinated a manual test of any new algorithms with radiologists before incorporating them and measured their effect via key performance indicators (KPIs)
Intervention characteristics			
Adaptability	While some aspects of the intervention were new for both Altarum and IA, the partner relationships and clinical environments were generally well known to both	Development of ImageSmart was a nascent experience for Altarum, which had done other work with these partners but never application development. Refinement of the tool resulted from a usability survey conducted by Altarum after initial launch of the tool	RadAdvisor was developed, tested, and implemented prior to use by IA
Workflows	Both Altarum and IA understood and valued the importance of tailoring the intervention to the existing workflow	Users of ImageSmart could eventually order imaging tests through the tool but were required to follow up on test results using the EHR	From a workflow standpoint, users of the RadAdvisor application were almost seamlessly linked to it from the EHR environment
Evidence strength and quality	Totally independently, Altarum and IA worked to create CDS based on accepted best practices drawn from existing guidelines	In terms of application development, Altarum worked closely with American College of Radiology (ACR) and American College of Cardiology (ACC) guidelines to translate these into a usable CDS tool, ImageSmart	IA’s partner, medCPU, an experienced CDS application developer, used an algorithm to combine guidelines from multiple sources
Trialability and compatibility	Elements of usability and user-centered design were important to both Altarum and IA and they worked to either build these in at the outset or address them in the implementation stages	Altarum solicited post-implementation feedback via a usability survey, which resulted in significant changes to ImageSmart. At launch, Altarum had to adjust to the limitations of the EHR context by initially providing access to ImageSmart through a separate provider portal rather than directly from the EHR. After noticing that adoption was low, Altarum was planning to provide EHR-specific access with its partners	medCPU incorporated an element of user-centered design through interaction with an advisory team lead by a radiologist from one of the Tenet hospitals to develop the final RadAdvisor application
Process of implementation			
Goal setting	As stated in their proposals, the goals of both Altarum and IA were to address cost and quality with radiology CDS	Addressing a clear return on investment as a barrier to adoption was a challenge for both awardees	Addressing a clear return on investment as a barrier to adoption was a challenge for both awardees
Champions	Altarum and IA worked hard to build internal champions both within and across their partner organizations	For Altarum, given the nature of its partnership arrangement, building an internal champion at every practice the innovation targeted was much more challenging	At IA, identifying and retaining key internal champions was hampered only by staff turnover
Engaging and executing	Though their approaches differed, Altarum and IA provided, either directly or through their partner organizations, critical training needed to use their CDS tools	Altarum relied heavily on its respective partners to provide the necessary training using a train-the-trainer model, with limited success. The use of ImageSmart was not required	IA and medCPU provided an ongoing in-person presence at the 4 Tenet hospitals for training and throughout rollout. The use of RadAdvisor was considered mandatory
Reflecting and evaluating	Both Altarum and IA understood the importance of and sought ways to act upon soliciting and responding to feedback throughout the design, development, and implementation processes	To solicit ongoing input on the development of ImageSmart, Altarum formed a high-tech steering committee. This effort also helped ensure the incorporation of local guidelines into ImageSmart. Altarum explored whether using ImageSmart would be helpful in getting imaging pre-approval, but the payer community knew nothing about the CDS, which did not change over the implementation period	Although IA and medCPU actively sought feedback on use of the RadAdvisor tool during their monthly visits and in meetings with ED directors, this meant often relying on the ED directors to be a “single voice” in gathering, reporting, and brainstorming solutions to issues. Turnover in the ED or lack of buy-in often resulted in suboptimal communication
Measurement capability and data availability	While the grant specification indicated some degree of evaluation be conducted, little else about the methods and measures was specified. Both Altarum and IA sought to plan and measure according to their long-term outcome goals	Altarum was more experienced with evaluations and focused on near-term measures of adoption and use followed by longer-term measures of patient impact. Altarum identified a key staff member to design and manage the evaluation including all data collection and reporting	IA focused almost exclusively on the ability to report on longer-term measures of patient impact and was largely unable to provide data on adoption and use. IA chose to handle data collection almost entirely electronically, with input from a subset of Tenet key stakeholders and providers, and provided reporting information through a dashboard and manual reports through the project manager
Measures of implementation			
Acceptability, adoption, and abandonment	Fundamentally, both Altarum and IA understood the need to develop and implement a technical solution that could ideally be seamlessly integrated into the traditional workflow to ensure clinical adoption	Altarum struggled with a weak technology contractor for UP to provide access to ImageSmart through its loosely-connected provider portal. Even when an EHR vendor was identified, get the EHR vendor’s attention was difficult because of the demand on their time to meet MU criteria. This meant a long, slow process for EHR or system integration of ImageSmart	Despite working closely with the Tenet hospital IT group to “append” its RadAdvisor service to the existing EHR in all 4 EDs, the initial launch of IA’s RadAdvisor was fraught with user interface issues, according to surveyed users. Working in concert with users, medCPU quickly adjusted and, in some cases, tailored changes for the ED environment
Sustainability	Organizationally, both Altarum and IA were committed to developing a CDS solution that would exist and be supported beyond the grant period	For Altarum, given the fact that so many EHR systems were in place across their provider practices, and multiple systems were using ImageSmart, much more planning was needed on their part to ensure successful ongoing adoption and use	Although using RadAdvisor required only minimal training, staff turnover and workflow changes required planned training opportunities after rollout
Reach and replicability	Part of the overall sustainability plans for both Altarum and IA were launching their CDS solution beyond the target markets for the grant. If successful, these would represent new business for both organizations	Altarum, adding a partner in MPP, demonstrated that ImageSmart could be effectively rolled out with another provider system as well. Altarum demonstrated that ImageSmart could be used for radiology CDS and cardiac imaging CDS and could launch a mobile app, but it had limited success in full EHR integration and lacked significant technical experience in developing CDS	IA demonstrated that RadAdvisor could be effectively rolled out at all 4 Tenet hospitals at different times. In terms of expanding RadAdvisor into other areas (eg, cardiac) and markets (other hospital systems), IA identified a strong key partner in medCPU and appeared well positioned to be successful in broadening the use of the tool
Penetration and evolvability	To drive adoption, both Altarum and IA considered the value of using incentives	Altarum did explore the use of incentives with only short-term and mixed results	IA did not explore the use of incentives to improve adoption and utilization. For IA, ED directors among the 4 Tenet hospitals saw the use of RadAdvisor as integral to their practice and did not think incentives would be appropriate. However, they did note that using incentives to obtain user feedback would have been helpful
Characteristics of individuals and teams			
Role, skills and competencies, and collective efficacy	Motivated to better ensure successful adoption, Altarum and IA worked diligently to develop or acquire the relevant skills to develop and implement their CDS innovations	Altarum, a non-profit research organization with a great deal of experience working with government contracts, had little to no experience developing and implementing radiology CDS tools. Altarum, a non-profit research organization, may have had different goals than a for-profit company would	In contrast, IA, a for-profit organization with a great deal of experience in working with radiology departments to deliver improved radiology services, was working with its new partner, medCPU, a technology company with sophisticated algorithm development expertise to develop RadAdvisor. As a for-profit organization, Imaging Advantage may have had different drivers for reaching its goals with this innovation
Outcomes			
Effectiveness and efficiency	Measures of success based on the desired outcomes were important to both Altarum and IA and were established and tracked to the best of their abilities	Altarum’s reports focused more on training, adoption, and utilization, and Altarum was developing methods to look at impact on provider behavior change and patient outcomes. Altarum was unable to look at adoption more discretely than at the practice level based on its data capture plans and the way the train-the-trainer model was implemented, because it did not necessarily ensure that providers themselves were trained at each practice (sometimes it was an office manager or another paramedical staff member)	IA reported based on KPIs developed in concert with hospital providers, focusing on reductions in inappropriate image orders, with some information on training, adoption, and utilization
Cost	Altarum and IA shared similar long term goals with the additional of partners, spending on the initiative, and commercialization strategy	Altarum identified a problem with a key partner early on in its technical development of ImageSmart which cost them both time and money. Given the time constraints of the award, perhaps Altarum should have acted sooner to identify a new partner	From a business standpoint, IA had the advantage of having experience with commercialization and was perhaps better positioned to envision what would be needed to sustain the innovation

### Outer setting

#### External networks

IA implemented its CDS innovation in the EDs of 4 Tenet Healthcare hospitals in the Chicago area. Altarum worked with 2 large outpatient practice partner organizations, MPP and UP with employed and independent physicians in Michigan. The settings of the 2 awardees required different approaches to many elements of the innovation, especially the implementation process.

#### Technical environment

Technical partners for IA (medCPU and Tenet hospitals IT) were well established, whereas the technical partners for Altarum engaged early on with UP but were less established, resulting in limited EHR integration for initial launch.

#### Clinical environment

The clinical partnerships for IA and Altarum were described as strong at the executive level, but with staff turnover (ED directors for IA) and weak links to clinicians (due in part to the decentralized model Altarum elected to use), retaining strong support for the innovation over time was more challenging. For IA, working within the Tenet hospital EDs provided a specific context for technical integration, training, and rollout. Altarum, because of the decentralized structure of its partner organizations (UP and MPP), struggled to find a willing and widely used EHR partner. Altarum was also challenged by choosing to work broadly in the diffuse and heterogeneous nature of its partners’ technical systems, as well as the less well-defined training and practice environments for their implementation.

### Inner setting

#### Implementation climate

IA and medCPU maintained a regular presence in the 4 Tenet hospitals to meet with users, observe and address problems, and field and respond to suggestions for enhancements or changes. In addition, with each change, IA and medCPU coordinated a manual test of any new algorithms with radiologists before incorporating them and measured their effect via key performance indicators (KPIs). Altarum largely used its partners to establish a connection with the provider end users of ImageSmart thereby inadvertently separating developers and end users.

### Intervention characteristics

#### Adaptability

Development of ImageSmart was a nascent experience for Altarum, which had done other work with its partners but never CDS or application development. Refinement of the tool resulted from a usability survey conducted by Altarum after initial launch of the tool. Conversely, RadAdvisor was developed, tested, and implemented prior to use by IA.

#### Workflows

From a workflow standpoint, users of the RadAdvisor application were almost seamlessly linked to it from the EHR environment. Users of ImageSmart could, with effort, order imaging tests through the tool but were required to follow up on test results using the EHR.

#### Evidence strength and quality

In terms of application development, Altarum worked closely with American College of Radiology (ACR) and American College of Cardiology (ACC) guidelines to translate these into a usable CDS tool, ImageSmart. IA’s partner, medCPU, an experienced CDS application developer, used an algorithm to combine and translate guidelines from multiple sources.

#### Trialability and compatibility

At launch, Altarum had to adjust to the limitations of the EHR context and lack of vendor support by initially providing access to ImageSmart through a separate provider portal rather than directly from the EHR. After noticing that adoption was low, Altarum was planning to provide EHR-specific access with its partners but these efforts would extend beyond the specified grant period. medCPU also incorporated an element of user-centered design through interaction with an advisory team lead by a radiologist from one of the Tenet hospitals to develop the final RadAdvisor application.

### Process of implementation

#### Goal setting

Although the main motivation for the CDS innovation was focused on improving efficiency, reducing inappropriate care, controlling cost, and preparing for new regulations for imaging selection (per the Merit-based Incentive Payment System/Medicare Access and Children’s Health Insurance Program Reauthorization Act), there has been no clear return on investment to providers. Addressing a clear return on investment as a barrier to adoption was a challenge for both awardees.

#### Champions

At IA, identifying and retaining key internal champions was hampered mainly by staff turnover. For Altarum, however, given the nature of its partnership arrangement, building an internal champion at every practice the innovation targeted was more challenging.

#### Engaging and executing

IA and Altarum provided training and education on their CDS innovations to relevant members of their partner organizations. IA and medCPU provided an ongoing in-person presence at the 4 Tenet hospitals for training and throughout rollout. Altarum relied heavily on its respective partners to provide the necessary training using a train-the-trainer model, with limited success. Although the use of RadAdvisor was considered mandatory, the use of ImageSmart was not required.

#### Reflecting and evaluating

Although IA and medCPU actively sought feedback on use of the RadAdvisor tool during their monthly visits and in meetings with ED directors, this meant often relying on the ED directors to be a “single voice” in gathering, reporting, and brainstorming solutions to issues. Turnover in the ED or lack of buy-in often resulted in suboptimal communication. To solicit ongoing input on the development of ImageSmart, Altarum formed a high-tech steering committee. This effort also helped ensure the incorporation of local guidelines into ImageSmart. Altarum explored whether using ImageSmart would be helpful in getting imaging pre-approval, but the payer community knew nothing about the CDS, which did not change over the implementation period.

#### Measurement capability and data availability

Despite matching goals and strong similarities in CDS development, training plans, and EHR integration, IA and Altarum differed in identifying ways to follow and report on key measures. Altarum was more experienced with evaluations and focused on near-term measures of adoption and use followed by longer-term measures of patient impact. IA focused almost exclusively on the ability to report on longer-term measures of patient impact and was largely unable to provide data on adoption and use. Altarum identified a key staff member to design and manage the evaluation including all data collection and reporting. IA chose to handle data collection almost entirely electronically, with input from a subset of Tenet key stakeholders and providers, and provided reporting information through a dashboard and manual reports through the project manager. Despite their parallel intended goals, the 2 awardees did not approach the capture of early, middle, and long-term measures the same way.

### Measures of implementation

#### Acceptability, adoption, and abandonment

Despite working closely with the Tenet hospital IT group to “append” its RadAdvisor service to the existing EHR in all 4 EDs, the initial launch of IA’s RadAdvisor was fraught with user interface issues, according to surveyed users. Working in concert with users, medCPU quickly adjusted and, in some cases, tailored changes for the ED environment. In contrast, Altarum struggled with a weak technology contractor for UP to provide access to ImageSmart through its loosely-connected provider portal. Even when an EHR vendor was identified, getting the EHR vendor’s attention was difficult because of the demand on their time to meet MU criteria. This meant a long, slow process for EHR or system integration of ImageSmart which had not yet been realized when the grant period ended.

#### Sustainability

Although using RadAdvisor required only minimal training, staff turnover and workflow changes required planned training opportunities after rollout. For Altarum, given the fact that so many EHR systems were in place across their provider practices, and multiple systems were using ImageSmart, much more planning was needed on their part to ensure successful ongoing adoption and use.

#### Reach and replicability

IA demonstrated that RadAdvisor could be effectively rolled out at all 4 Tenet hospitals at different times. Altarum, adding a partner in MPP, demonstrated that ImageSmart could be effectively rolled out with another provider system as well. IA and Altarum had plans to expand to other markets with their radiology CDS tools. In terms of expanding RadAdvisor into other areas (eg, cardiac) and markets (other hospital systems), IA identified a strong key partner in medCPU and appeared well positioned to be successful in broadening the use of the tool. Altarum demonstrated that ImageSmart could be used for radiology CDS and cardiac imaging CDS and were planning the development and launch of a mobile application but had limited success in full EHR integration and lacked technical experience in developing CDS.

#### Penetration and evolvability

IA did not explore the use of incentives to improve adoption and utilization. Altarum did explore this with only short-term and mixed results. For IA, ED directors among the 4 Tenet hospitals saw the use of RadAdvisor as integral to their practice and did not think incentives would be appropriate. However, they did note that using incentives to obtain user feedback would have been helpful. Altarum and IA leveraged the impending Medicare 2017 requirement to use CDS for radiology ordering, but this had limited impact as an adoption incentive. Both awardees talked with payers about the possibility that pre-authorizations might be eliminated by using effective CDS, but these talks did not produce any resulting policy changes.

### Characteristics of individuals and teams

#### Role, skills and competencies, and collective efficacy

Altarum and IA identified critical clinician early adopters to help “evangelize” the CDS tool within each of the implementation environments. IA and Altarum reported being able to maintain staffing levels without concern over losing grant funding at the end of the project period. Altarum, a non-profit research organization with a great deal of experience working with government contracts, had little to no experience developing and implementing radiology CDS tools. In contrast, IA, a for-profit organization with a great deal of experience in working with radiology departments to deliver improved radiology services, was working with its new partner, medCPU, a technology company with sophisticated algorithm development expertise to develop RadAdvisor. As a for-profit organization, IA may have had different drivers for reaching its goals with this innovation than Altarum, a non-profit research organization. However, at the end of the grant funding period, both awardees indicated that their innovations would be sustained beyond the funding period and that use would extend beyond their existing partners.

### Outcomes

#### Effectiveness and efficiency

Although their reporting methods and goals differed somewhat, IA and Altarum provided monthly reporting information to their partners. IA reported based on KPIs developed in concert with hospital providers, focusing on reductions in inappropriate image orders, with some information on training, adoption, and utilization. Altarum’s reports focused more on training, adoption, and utilization, and Altarum was developing methods to investigate impact on provider behavior change and patient outcomes. In both cases, reports on utilization were used among a subset of providers to encourage compliance with the CDS tools. Although both awardees may have intended to address issues of adoption and use of their CDS imaging tools, it was not possible to track this metric based on the measures IA targeted for data collection. In addition, Altarum was unable to look at adoption more discretely than at the practice level. This resulted from its data capture plans and the way the train-the-trainer model was implemented, which did not necessarily ensure that providers themselves were trained at each practice (sometimes it was an office manager or another paramedical staff member).

#### Cost

Altarum encountered a problem with a key partner early on in its technical development of ImageSmart which cost them both time and money. Given the time constraints of the award, Altarum needed to act quickly to identify a new partner. Altarum and IA pledged to show significant decreases in the costs associated with inappropriate image orders, which was hard to do in the time frame mandated by the award. From a business standpoint, IA had the advantage of having experience with commercialization and was perhaps better positioned to envision what would be needed to sustain the innovation. A downstream goal of these innovations was to demonstrate clinical improvement, but this was hard to track given the poor definition for near-term measures in the case of IA and poor provider adoption in the case of Altarum.

## DISCUSSION

Altarum and IA developed strikingly similar CDS tools, implemented them in contexts that differed considerably in complexity, and achieved different levels of successful adoption. While contextual differences contributed to challenges in ensuring successful adoption, these issues have been well studied and key design decisions could have adequately addressed them. Importantly, other factors were at play as well. IA ensured clinical adoption of their radiology CDS by controlling for complexity in choosing to embed RadAdvisor fully into the EHR environment in the Tenet hospital EDs involved in the innovation. By bringing a wealth of experience to the health IT challenges in terms of their own experience working with radiologists and by selecting an experienced IT partner, IA mitigated risk for the complex ED environment. In addition, IA and its partners understood that the busy, complex, clinical environment they would be seeking clinical adoption in would require a carefully planned and executed implementation strategy to ensure adoption.

These factors, managing for the complexity of implementation (eg, type and number of EHR systems), prior experience with congruent innovations, and mitigating risk due to the diversity of clinical settings, worked together to influence provider adoption of CDS tools. Health care professionals aiming to implement CDS and ensure adoption should consider how managing factors in the implementation context is critical to their success.

### Addressing implementation complexity

While both IA and Altarum where very familiar with the clinical environments they chose to work in, they handled those complexities unequally. IA prepared a tool to work in a group of 4 EDs under the same organizational umbrella, all using 1 EHR system. Altarum built a tool to work across multiple outpatient clinical practices, which were loosely affiliated with one another and used a variety of EHR systems. For Altarum, both technical partner issues and the relative complexity of the outpatient clinical practice environment introduced adoption challenges. Despite well laid plans, ultimately, Altarum’s users were forced to use a third portal or intermediary system and the necessity of this intermediary system was likely the single largest contributor to poor adoption of the tool.

### Clinical diversity

Research shows that overall user experience and adoption likelihood are tied to seamless workflow integration.^19^ IA chose to design a tightly coupled solution and work hard to implement it in a specific setting. More than 27% of the Altarum providers who responded to our 2015 provider survey described their tool as “somewhat hard to use,” and 4% described it as “very hard to use.” In contrast, none of the IA providers described their tool as hard to use.

### Stakeholder experience

Experience of the awardees and their partners was key to laying the groundwork for adoption. Although neither Altarum nor IA had experience implementing CDS tools in the sites selected for the HCIA project, IA had previously set up teleradiology services in the hospitals where its CDS was deployed. This experience and familiarity helped project leaders better anticipate challenges and facilitators of adoption, including dealing with time constraints, altering workflows, mandating use, and connecting users with developers. IA’s choice to eliminate obstacles like optional use and multiple sign-ons facilitated CDS adoption, even though the workflow was more complicated. Experience with CDS tools and related innovations are particularly important in clinical settings with diverse technological systems and geographically distributed users.

Experienced vendors can facilitate adoption by helping organizations avoid or address barriers to use. IA’s seasoned technical vendor developed a CDS tool that worked seamlessly with the EHR system, allowing easier movement between the application and the patient record, thereby encouraging (or not discouraging) providers to use the tool. The experienced vendor also provided on-site support to answer questions and address concerns, provide training, and do problem troubleshooting at all sites using the tool. Altarum and its less experienced partners used a train-the-trainer model with limited results.

CDS tools are designed to help health care providers deliver the most appropriate care at the right time.^20^ Moving from clinical practice guidelines to evidence-based CDS tools remains complicated. Guidelines may not be written in ways that can be practically implemented during clinical encounters. Organizations designing CDS tools must take certain liberties in interpreting professional guidance and making it relevant for provider use, especially when tools will be used in generalist and specialty outpatient settings. Generalist and specialty providers serve different types of patients with varying needs, making it difficult to accommodate everyone with a single interpretation and implementation of the guidelines. Altarum’s users and patients reflected such diversity, whereas IA’s users and patients more homogenous. Altarum’s users needed a tool suitable for a wide range of clinical encounters therefore Altarum needed to invest more time in interpreting and tailoring than IA did.

### Strengths and limitations

While the CFIR is generally held to be a strong tool for making cross-context comparisons, the contextual differences observed in this comparative case study may limit the generalizability of these findings to other interventions. However, much of the evidence on the use of CFIR suggests that it is robust for such comparisons. Limitations of the CFIR for this research may have included insufficient constructs to address scale-up, spread, and sustainability, a limited ability to capture information about constructs over time, and a lack of a complete picture for the pre, during, and post organizational capacity and needs. In addition, the CFIR does not really connect concretely to outcomes or mandate or marshal the collection of data for specific outcomes. The CFIR is also best suited to a prospective analysis that is completely integrated into the implementation process.

### Implications

It is rare to see 2 distinct groups attempt such similar initiatives within the same funding instrument, therefore, it was compelling to look back and see what went right and what went wrong for the 2 innovations. Moreover, because of the similarities between these 2 initiatives, it was important to investigate their comparative differences (context notwithstanding). Application of the CFIR framework post hoc as a tool for comparison provided some key insights into their relative successes and failures. For CDS innovations, complex implementation contexts, defined as the number of implementation sites and EHR systems, create barriers to adoption and ease of tool use. However, these challenges are already fairly well understood and can be addressed with appropriate implementation design. Similarly, stakeholders’ experience with developing and implementing HIT tools has a direct impact on adoption, especially as complexity increases. Planning for the diverse clinical contexts in which CDS tools will be used should be guided by decisions that mitigate risk, address the complexity challenges, and tailoring to facilitate adoption.

Other factors may have been at work as well, for example, the drivers for adoption of EHRs have more recently been shown to be unrelated to direct support of patient care and to insufficiently take into account the needs of the provider.^21^ In addition, in these innovations there appears to be a gap between the extant published literature, which clearly shows evidence on the negative impact of common mistakes on provider adoption, and the limited methods used by these 2 awardees to ensure adoption. Perhaps part of this is due to the delay between research and practice, but it may also be due in part to the limited experience of these awardees.

## CONCLUSION

Sufficient review of the existing literature on CDS implementation and adoption should be undertaken in advance of designing an intervention. This is even more important in the face of complex environments and/or inexperienced implementers. CDS has been shown to generally have an indirect impact on long-term effects like reducing cost, improving quality, and improving patient outcomes, if any, and to do so over longer periods of time. These innovations are likely to be more successful from the outset if funding agencies require more clarity up front about overall implementation plans. This should include the methods to address the established success criteria and the details on how data collection will be executed to support evaluation.

Some of the factors observed over the course of this evaluation may have been affected by the overall maturity of the implementation of these innovations. Both awardees were planning to continue their radiology CDS work and even expand into other geographic areas. Revisiting this assessment as these CDS innovations mature may provide additional insights.

## FUNDING

This publication was made possible by Contract Number HHSM-500-2010-00021I from the U.S. Department of Health and Human Services, Centers for Medicare & Medicaid Services. The contents of this publication are solely the responsibility of the authors and do not necessarily represent the official views of the U.S. Department of Health and Human Services or any of its agencies.

## Author contributors

All authors listed meet the ICMJE criteria for authorship for this manuscript. Specifically, LHM and DSJ served as subject matter experts for the evaluation of these 2 awardees and were involved in interviewing, protocol development, survey development, and made significant authoring and revising contributions to the manuscript. MRS was responsible for data analysis and interpretation for these 2 awardees and contributed to authoring and revising the manuscript. SRJ was responsible for overall execution of the provider survey (across the entire evaluation) and contributed to authoring and revising the manuscript. BB and LRS served as subject matter experts in CDS and CER/CFIR respectively (across the entire evaluation, led by LRS) and both contributed to authoring and revising the manuscript. All authors gave their approval for the final version to be published.

## SUPPLEMENTARY MATERIAL

Supplementary material is available at *Journal of the American Medical Informatics Association* online.

*Conflict of interest statement*. None declared.

## Supplementary Material

Supplementary Data
