# Supplementary material for: A qualitative framework-based evaluation of radiology clinical decision support initiatives: eliciting key factors to physician adoption in implementation
Source: JAMIA Open. 2019 Feb 22;2(1):187–96. doi: 10.1093/jamiaopen/ooz002 (PMC6952024; doi:10.1093/jamiaopen/ooz002)
Supplement: Supplementary Data [file ooz002_supp.docx]

**Appendix A: List of organizations and abbreviations:**

- CDS = clinical decision support
- EHR = electronic health record
- HIT = health information technology
- CMMI=Center for Medicare and Medicaid Innovation
- HCIA=Health Care Innovation Awards
- Altarum Institute (Altarum): HCIA awardee implementing radiology CDS intervention in physician practices
  - Maclaren Physician Partners (MPP): outpatient practice partner organization to Altarum
  - United Physicians (UP): outpatient practice partner organization to Altarum
- Imaging Advantage (IA): HCIA awardee implementing radiology CDS intervention in emergency departments
  - ImageSmart: radiology CDS application used by Altarum’s partners and a ‘home grown’ application developed by Altarum)
  - Tenet Healthcare: Health system providing EDs for IA innovation
  - MedCPU: IT partner for IA innovation RadAdvisor
  - RadAdvisor: radiology CDS application used by IA’s partners and developed by MedCPU

**Appendix B: Sample provider survey questions**

1. **Do you provide direct medical care services to patients as part of [innovation]?***Those providing direct medical care may include, but are not limited to physicians, nurses, advanced clinic providers, counselors, pharmacists, EMTs, and therapists.*

- Yes
- No
- Unknown

1. **Were you at your practice prior to implementation of [innovation] in [date]?**

- Yes
- No

1. **Overall, how familiar are you with [innovation]?**

- Extremely Familiar
- Moderately Familiar
- Somewhat Familiar
- Slightly Familiar
- Not at all Familiar

1. **In what ways have you been involved with [innovation]?**

- Leader/Champion that oversees implementation
- Direct involvement (For example, I am the end user of the health IT innovations)
- Indirect involvement (For example, I do not work directly with the health IT innovations, but members of my staff or my colleagues do and/or I receive information from those that are using the innovations)
- No involvement with this innovation
- Other—*Please specify:* ________________________________________________

1. **Overall, has the way you care for patients been impacted by the implementation of [innovation]?**

- Yes – Go to 5a
- No – Skip to 6

**5a. If yes, has [innovation] had a positive or negative impact on the care of your patients?**

- Very positive
- Somewhat positive
- Neither positive nor negative
- Somewhat negative
- Very Negative

**Provider Satisfaction:**

This next section of this survey will ask questions about your satisfaction with [innovation].

1. **How satisfied are you with [innovation] overall?**

- Extremely Satisfied
- Very Satisfied
- Moderately Satisfied
- Slightly Satisfied
- Not at all Satisfied

1. **Please indicate how easy or hard it is to use [innovation] as it has been implemented.**

- Very Easy to Use
- Somewhat Easy to Use
- Neither Easy nor Hard
- Somewhat Hard to Use
- Very Hard to Use
- Not Applicable

1. **Based on the functionality of [innovation], please indicate if your system has done each of the following.**

|  | **Yes, Within the Past 30 Days** | **Yes, but Not Within the Past 30 Days** | **No, Not at All** | ***Not Applicable*** |
| --- | --- | --- | --- | --- |
| 1. Alerted me to a potential medication error |  |  |  |  |
| 1. Reminded me to provide preventive care (e.g., vaccines) |  |  |  |  |
| 1. Helped me order fewer tests due to better availability of lab/radiology results |  |  |  |  |
| 1. Facilitated direct communication with a patient (e.g., email or secure messaging) |  |  |  |  |
| 1. Alerted me that I received a patient summary from another provider |  |  |  |  |
| 1. Led to some unintended consequences (e.g., “alarm fatigue” or incorrect identification of potential problems) |  |  |  |  |

1. **Please indicate how much you agree or disagree with each of the following statements regarding the potential impacts of [innovation].**

|  | **Strongly Agree** | **Somewhat Agree** | **Neither Agree nor Disagree** | **Somewhat Disagree** | **Strongly Disagree** | | ***Not Applicable*** |
| --- | --- | --- | --- | --- | --- | --- | --- |
| 1. [innovation] helps me provide better patient care |  |  |  |  |  | |  |
| 1. [innovation] leads to more effective communication during patient visits |  |  |  |  |  | |  |
| 1. [innovation] has improved my patients’ access to care |  |  |  |  |  | |  |
| 1. [innovation] helps me develop good relationships with my patients |  |  |  |  |  | |  |
| 1. Overall, my practice functions more efficiently with [innovation] |  |  |  |  |  | |  |
| 1. [innovation] saves me time |  |  |  |  |  | |  |
| 1. [innovation] has increased the time I am able to spend with patients during office visits |  |  |  |  |  | |  |
| 1. [innovation] has increased the average number of weekly office visits to my practice |  |  |  |  |  | |  |
| 1. Sufficient resources (e.g., support staff, time, training) have been provided for me to use/interact with [innovation] |  |  |  |  |  | |  |
| 1. [innovation] produces financial benefits for my practice |  |  |  |  |  | |  |
| 1. Investing in [innovation] is worthwhile in terms of time, energy, and resources |  |  |  |  |  | |  |
|  | **Strongly Agree** | **Somewhat Agree** | **Neither Agree nor Disagree** | **Somewhat Disagree** | **Strongly Disagree** | | ***Not Applicable*** |
| 1. [innovation] prevents me from providing high quality patient care |  |  |  |  |  | |  |
| 1. The added logistics required by [innovation] (i.e., paperwork, administration) is a burden on me and/or my staff |  |  |  |  | |  |  |
| 1. [innovation] has improved perceived patient satisfaction with care |  |  |  |  | |  |  |
| 1. Sufficient technical IT support is available to operate [innovation] |  |  |  |  | |  |  |
| 1. [innovation] has been integrated into clinical workflow |  |  |  |  | |  |  |

Questions also addressed clinical care workflow, timeliness of care, patient care quality, and basic demographic information.

**Appendix C: Sample condensed interview guide**

**Respondent Background:**

1. What’s your role? How long have you been involved? How has your role changed?

**Innovation:**

1. Please describe the main components of the innovation and for each of those components what [awardee], [partner], and other partners are doing?
   1. Probe on 4 areas
      1. CDS and HIE
         1. How do “alerts” for image study reports work for HIE?
      2. Patient education program and targeted care management (alternative care)
      3. Provider outreach, education, & incentive program
      4. Track and evaluate patient outcomes
   2. Probe on dose, reach, fidelity
   3. How do you target providers? How do you educate patients?
   4. Is data used (i.e. utilization reports, dashboards) by [awardee], [partner], others to support innovation goals?
2. What changes (to the innovation), if any? (From the application, from operational plan). Reasons for changes? “Unplanned adaptations?”
   1. Incentives?
   2. Partner changes? EHR vendors – systems integration?
   3. Target imaging (CT and MRI Lumbar & spine)?; new imaging (cardiac)?
3. Which components are critical to the success of this innovation? / What aspects of your innovation model have or will have the most impact on improving appropriate imaging, reducing costs, etc?
4. Have you/or your organization ever implemented an innovation like this before? Please describe your prior experience.

**Appendix D: Sample coded interview passages**

These passages were coded for ‘implementation process’:

I:

Regarding staffing, you indicated that the resources specifically dedicated to [EHR] integration were brought on board and a project manager to replace someone who left, and a QI analyst and internal medicine trainee. Is that one in the same role?

R:

No, those are two separate roles for QI and the other is role of physician engagement. He’s also played a key role related to enhancing the best practice guidelines and looking for gaps in content. One of the things that came out of our user interviews and certainly part of the mid-documentation demonstration project is after a year of looking at the data and finally getting the users online, there are still some significant content gaps not being provided from the professional society. That is a key barrier to use as well as an opportunity for us to try to figure out how to address those barriers and help physicians through the process when what they are looking for isn’t there.

I:

What components are critical to the success of the innovation?
R:

Success as of today or what it means long term?
I:

Both.
R:

I think being able to interact with multiple work flows and being able to support it---it will be a long time before Clinical Decision Support is built into every EHR. There are some top players, but you still need to have some viable options because there’s not just one size fits all for organizations. You need web-based, you need mobile, and I think having multiple avenues to support those multiple workflows is critical. I think having full content is the next area. Outside of the two organizations that we have content from it is important to build that out to make sure users have access. We had to use the content and get feedback and see what was missing and we submitted requests to the ACR and did not get responses. I think the capabilities for localization is the other area. The professional societies, they are really focused on recommendations, that’s what they do. You need to make sure you have the partners and the clinical input outside of radiology to make sure the recommendation is complemented with practical application of it. Then the other key factor is patient engagement and shared decision making.
